# A dearth of specifications regarding primary diagnostic monitors (PDMs) for nuclear medicine leaves us with little guidance during the COVID‐19 pandemic

**DOI:** 10.1002/acm2.12949

**Published:** 2020-06-15

**Authors:** Na Song, Lionel S. Zuckier

**Affiliations:** ^1^ Division of Nuclear Medicine Department of Radiology Montefiore Medical Center and Albert Einstein College of Medicine Bronx NY USA

Dear Editor,

During the current coronavirus COVID‐19 pandemic, remote interpretation of medical images, such as viewing studies from home, has become imperative for facilities to achieve their social‐distancing mandate and provide a safe workplace for image interpreters. To insure accurate interpretation of studies, it is self‐evident that monitors used for image interpretation, which we will refer to as primary diagnostic monitors (PDMs), must achieve adequate quality assurance standards and be compliant with relevant regulatory requirements.

In spite of this, almost all published regulations regarding PDMs do not provide clear instructions specific to the interpretation of nuclear medicine images and appear to aggregate all PDMs into the same set of specifications; according to our thinking this is not a reasonable alternative. The reports from the American Association of Physicists in Medicine (AAPM) Task Group 18 (TG18)^1^ and the ACR–AAPM–SIIM Technical Standard for Electronic Practice of Medical Imaging^2^ are all pertinent to performance of higher‐resolution modalities such as mammography and diagnostic CT but do not directly address needs specific to nuclear medicine. Most recently, the AAPM, in an online resource entitled “COVID‐19 Information for Medical Physicists,” shared a procedure note authored by the Clinical Imaging Physics Group at the Duke University School of Medicine which provides helpful guidance in setting up a temporary display device for diagnostic interpretation. Once again, this communication detailed high‐resolution monitor specifications and neither excluded nor addressed nuclear medicine needs.^3^ Tellingly, the NYC Department of Health and Mental Hygiene Office of Radiological Health, which licenses our facility, has recently revised guidelines regarding quality assurance programs for diagnostic facilities in New York City and, based on comments from users, explicitly excluded nuclear medicine imaging from the requirements for PDMs, Although not substituting replacement standards.^4^

The main source of specific guidance regarding nuclear medicine monitors that we have identified is authored by the AAPM Task Group 177 which states that “In the absence of any definitive study to determine the display characteristics needed for nuclear medicine monitors used for image interpretation, the task group recommends that nuclear medicine monitors should have a display white (maximum luminance) >120 cd/m^2^, minimum luminance for black < 2 cd/m^2^, and luminance nonuniformity of <20%.”^5^ In terms of quality assurance, the SNMMI and European Association of Nuclear Medicine (EANM) have authored a Practice Guideline for Tele‐Nuclear Medicine 2.0, which notes that full quality control of a monitor is not practical for remote sites, but states that “relatively simple test patterns should be readily displayable.”^6^

In our opinion, the need to codify and publicize specific standards and practical guidance regarding nuclear medicine remote reading stations and monitors on a national level is pressing. Even in the current situation, institutions may be loath to rely on home workstations and monitors that do not meet explicitly approved standards; the alternative of deploying monitors that are compliant with unnecessarily strict imaging standards would be wasteful if not impossible in situations of constrained resources and increased demand. As suggested in the SNMMI and EANM guidelines, it may be appropriate to allow some leniencies regarding off‐site monitors as a practical matter. Perhaps this would be an opportune time for the SNMMI to issue updated guidelines related to remote viewing of nuclear medicine images both in routine and exigent situations. It is our hope that the situation of expanded reading of nuclear medicine studies outside of the hospital engendered by the COVID‐19 pandemic may bring this need to the surface and galvanize some degree of change.

## References

[1] Samei E , Badano A , Chakraborty D , et al. Assessment of display performance for medical imaging systems: executive summary of AAPM TG18 report. Med Phys. 2005;32:1205–1225. 15895604 10.1118/1.1861159

[2] Norweck JT , Seibert JA , Andriole KP , et al. ACR‐AAPM‐SIIM technical standard for electronic practice of medical imaging. J Digit Imaging. 2013;26:38–52. 22992866 10.1007/s10278-012-9522-2 PMC3553359

[3] Setting Up A Temporary Display Device. https://www.aapm.org/AAPMUtilities/download.asp?file=COVID19/Setting%20Up%20A%20Display%20Device.docx&cstkey=2f04bb8e-c6a6-460c-9672-f8a182446ad6. Accessed Mar 24, 2020.

[4] New York City Health Code. Article 175 – Radiation Control §175.12 Quality Assurance (QA) program requirements for diagnostic facilities. https://www1.nyc.gov/assets/doh/downloads/pdf/about/healthcode/health-code-article175.pdf. 6577360

[5] Halama JR , Graham D , Harkness BA , et al. AAPM Task Group 177: Acceptance Testing and Annual Physics Survey Recommendations for Gamma Camera, SPECT, and SPECT/CT Systems. AAPM; 2019.

[6] Parker JA , Christian P , Jadvar H , Sattler B , Wallis JW . The SNMMI and EANM practice guideline for tele‐nuclear medicine 2.0. J Nucl Med Technol. 2014;42:15–19. 24375155 10.2967/jnmt.113.133231

